# Loop-mediated isothermal amplification (LAMP) and Polymerase Chain Reaction (PCR) as quality assurance tools for Rapid Diagnostic Test (RDT) malaria diagnosis in Northern Namibia

**DOI:** 10.1371/journal.pone.0206848

**Published:** 2018-12-12

**Authors:** Munyaradzi Tambo, Mary Mwinga, Davis R. Mumbengegwi

**Affiliations:** 1 Multi-Disciplinary Research Centre, Science Technology and Innovation Division, University of Namibia, Windhoek, Namibia; 2 Department of Biological Sciences, University of Namibia, Windhoek, Namibia; Academic Medical Centre, NETHERLANDS

## Abstract

Malaria cases sometimes go undetected using RDTs due to their inaccurate use, poor storage conditions and failure to detect low parasitaemia (<50parasites/μL). This could result in continuous transmission of malaria and sustenance of parasite reservoirs. Molecular diagnostic tools are more sensitive and specific than RDTs in the detection of *plasmodium* parasites. However, the Polymerase Chain Reaction (PCR) is not routinely used because equipment and reagents are expensive and requires highly skilled personnel. Loop-mediated isothermal amplification (LAMP) is a relatively new molecular diagnostic tool for malaria with all the advantages of PCR (sensitive and specific) without the mentioned disadvantages. However, it has not been evaluated extensively as a point of care diagnostic in the field. One hundred and fifteen used RDTs were collected from health facilities in Northern Namibia in a blind study and PCR and LAMP were used to determine the presence of *Plasmodium* DNA. The sensitivities and PPV were 40.91% and 90% respectively for RDTs, 72.73% and 100% respectively for PCR with LAMP as the golden standard. In low malaria transmission settings, LAMP can be also be considered for use as a surveillance tool to detect all sources of malaria and determine proportion of low parasitaemia infections in order to eliminate them.

## Introduction

Malaria remains a global public health concern with 92% of the mortality burden in Africa [1]. The negative impact of malaria on social and economic structures has made it a focal point of the international development agenda. Therefore goals have been set world-wide to scale up malaria control in order to achieve global malaria eradication [2]. Malaria is one of the major public health concerns in Namibia although it is both treatable and preventable [3]. World Health Organization (WHO) recognizes 17 countries including Namibia as having pre-elimination or elimination programs [4]. In Namibia malaria transmission is confined to the North eastern and north central parts where it is endemic. However, malaria has declined from 477 000 in the year 2000 to about 4745cases in 2014 and then plateaued till 2017 [5]. Therefore the National Malaria Control Program (NMCP) adopted a strategy in 2010 to eliminate malaria from Namibia by the year 2020, now adjusted to 2023 [6].

A recent study estimated that in low transmission settings, sub-patent infections comprise the majority (70–80%) of all malaria infections [7; 8]. Therefore in a low transmission setting like Namibia, there could be a significant number of sub-patent infections. These are infections with low parasite density; where infected individuals do not show symptoms of malaria. If these sub-patent infections are not identified there is a risk of onward transmission of malaria defeating the efforts to eliminate malaria in Namibia. Currently the diagnosis of malaria in Namibia is by use of the CareStart Rapid Diagnostic Tests (RDTs) as per WHO recommendation, which detect the PfHRP2 and pLDH antigens of the *Plasmodium* parasite [9; 10]. In Namibia, the CareStart RDTs are routinely to test for malaria by nurses as they are suitable for field use (portable cassette that does not require electricity). The CareStart RDTs are lot tested by the WHO program for quality control. However, some studies have shown RDTs are not sensitive enough to detect asymptomatic malaria cases which are usually at low parasite density that is below 50parasites/μl [11; 12; 13]. Since Namibia is a low transmission setting it is possible that the majority of infections are missed by these RDTs. In addition, persistence of antigens (Histidine Rich Protein-2) in the body for up to four weeks after treatment (parasite clearance) gives rise to false positives [14]. Microscopy, the golden standard for malaria has also been reported to miss cases at low parasite density, is time consuming and often subject to unreliable results from different laboratories [15; 16]. In order to reduce the risk of mortality and/or severe disease, rapid and accurate diagnosis is of utmost importance [17].

Molecular methods such as the Polymerase Chain Reaction (PCR) and Loop-mediated isothermal amplification (LAMP) have shown promise in diagnosis of malaria in low transmission settings where low density infections missed by RDTs could sustain infection [18; 19]. The high sensitivity is as a result of the amplification of the signal for detection (parasite DNA) by these molecular techniques. The two techniques are DNA amplification tools that differ in the way that they amplify the DNA. PCR uses a thermo-cycler to amplify DNA using different temperatures for denaturing and annealing, it has a detection threshold of 1-2parasites/μL. On the other hand LAMP amplifies DNA through strand displacement at a single temperature of 65°C with a detection threshold of 1-2parasites/μL [20]. Currently, nested PCR is the validated golden standard but LAMP has been reported to be as sensitive and in other cases more sensitive than PCR in a number of studies [21; 22]. The use of tools like LAMP and PCR may certainly help identify remaining “hot spots” and “hot pops” of malaria infections which can be dealt with using appropriate interventions. This study used the advantages of specificity and sensitivity of PCR and LAMP, to use them as evaluation and quality assurance tools to make sure all malaria cases including the sub-patent infections are identified. In this study, LAMP was used as the golden standard and not PCR because LAMP has been reported to be more sensitive than PCR as its sensitivity is not affected by left over hemoglobin like PCR [22; 23; 24].

## Methods

The work conducted under this study received ethical clearance from the Ministry of Health and Social Services Namibia’s Biomedical Research and ethics committee. The target population of the study was adult RDT tested individuals from Northern parts of Namibia who presented with malaria symptoms at health facilities. A total of 115 RDTs that had been used for testing for malaria by nurses at health facilities in the Ohangwena and Omusati regions of Namibia were supplied by the Ministry of Health and Social Services. The collected RDTs were the source of DNA for both PCR and LAMP diagnosis. The work conducted under this study received ethical clearance from the Ministry of Health and Social Services Namibia’s Biomedical Research and ethics committee.

### DNA extraction

DNA was extracted from a total of 115 RDTs that had been used for testing for malaria by nurses at Health facilities. The Chelex method was used to extract DNA from the RDTs. The method involves addition of 50 μl of saponin in 1000 μl of Phosphate Saline Buffer (PBS) to the RDT strip and incubating overnight at 4°C to remove the hemoglobin which is a PCR inhibitor. After the incubation period, the solution is aspirated and samples are washed with 1000μl PBS buffer that is aspirated and discarded after 30 minutes of incubation. A total of 100μl of nuclease free water and 50μl of 20% chelex solution is added to the samples and then heated at 98°C for 10 minutes to lyse the cells. The Chelex binds to the metals from the cells which could inhibit the PCR reaction. The Chelex is after heating by aspirating 100μl of the eluted DNA and transferring it into a new pre-labeled tube, care is taken to not aspirate any chelex beads. The extracted DNA is stored at -20°C for use in further analysis.

### PCR

**Fig 1:**
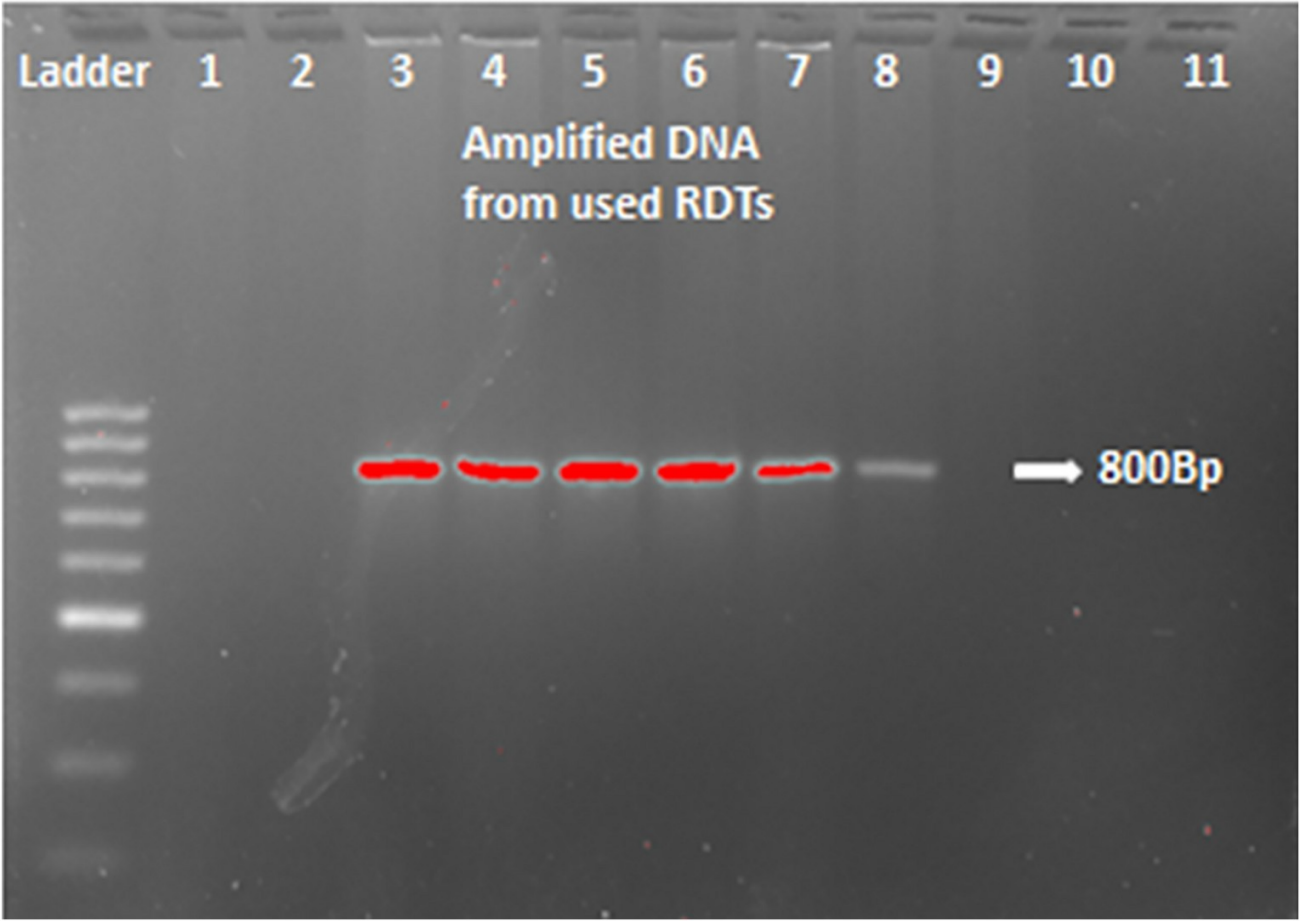
A gel showing PCR amplicons in the form of bands, indicating malaria infection. Lane 1- positive control, lanes 2 & 11- negative control, lanes 3, 4, 5, 6, 7, & 8 are positive samples, lanes 9 & 10 are negative samples.

PCR was performed with pan primers Plasmo 1(5’GTTAAGGGAGTGAAGACGATCAGA 3’) and Plasmo 2 (5’AACCCAAAGACTTTGATTTCTCATAA 3’) targeting the ssRNA gene in order to amplify *Plasmodium* DNA in order to determine if there was a malaria infection based on presence or absence of *Plasmodium* DNA. PCR was performed on all 115 samples with 5μl of DNA template extracted from the RDTs. The conditions were as follows: Initial denaturation 94°C for 2 min, 36 cycles 94°C for 45 sec, 56°C for 45 sec, 72°C for 2 min and hold at 4°C. The PCR products were run on a 2% agarose gel at 90 volts for 90 minutes. After gel electrophoresis, the visualization of results was done under Ultra Violet (UV) light (Fig 1).

### LAMP

LAMP was performed to amplify Plasmodium DNA to determine if there was *Plasmodium* DNA (indicator of malaria infection) in the blood samples. LAMP was run on the same 115 DNA samples used for running PCR. A total of 15μl of DNA template was mixed with pan primers (FIP and BIP comprising F1, F2 and B1, B2 priming sites, correspondingly and two “Displacement primers” F3 and B3) and the *Bst* polymerase (from *Bacillus stearothermophilus*) that comes dried in the LAMP tube caps [20]. The LAMP tubes were inverted 5 times to mix the DNA template with the reagents in the LAMP tube cap. A thermo-cycler was used to amplify DNA at 65°C for 45 minutes. Visualization of the results was by comparing change in turbidity using the negative and positive controls as references by fluorescence under UV light on a table top.

## Results

### Rapid diagnostic tests

A total of 10 RDTs (8.7%) were positive from the 115 samples collected from clinics in the field. RDTs detected a significantly lower number of malaria infections compared to LAMP (Table 1).

**Table 1:** RDT vs LAMP diagnostic efficacy.

**Difference**	10.44%
**95% CI**	0.9298 to 19.9331
**Chi-squared**	5.211
**DF**	1
**Significance level**	P = 0.0224

### Polymerase Chain Reaction (PCR)

A total of 16 samples were positive by PCR indicating malaria infections, PCR had 6 more positives than RDTs. Detection of malaria infections was not significantly different between PCR and RDTs (Fig 1 &Table 2). However, PCR detected more infections than RDTs.

**Table 2:** PCR vs RDT diagnostic efficacy.

**Difference**	5.22%
**95% CI**	-3.6167 to 14.1067
**Chi-squared**	1.556
**DF**	1
**Significance level**	P = 0.2122

### Evaluation of diagnostic performance of RDTs and PCR

A diagnostic evaluation test was performed to compare RDTs and PCR with LAMP as the golden standard for the diagnosis of malaria. The sensitivity of RDTs was about half that of PCR and less than half the sensitivity of LAMP (Tables 3 & 4).

**Table 3:** Diagnostic evaluation testof RDTs and PCR with LAMP as the golden standard.

Statistic	Sensitivity % (95% CI)	Specificity % (95% CI)	PPV % (95% CI)	NPV % (95% CI)
RDT	40.91 (20.71–63.65)	98.92 (94.15–99.97)	90 (54.59–98.54)	87.62 (83.32–90.95)
PCR	72.73 (49.78–89.27)	100 (96.11–100)	100	93.94 (88.68–96.84)
LAMP	Reference	Reference	Reference	Reference

**Table 4:** Diagnostic perfomance of PCR vs RDTs with LAMP as the golden standard.

Diagnostic tools	Number of true positives	Number of true negatives	Number of false positives	Number of false negatives
CareStart Rapid Diagnostic Test (RDT)	9	93	1	12
Polymerase Chain Reaction (PCR)	16	93	0	6
Loop-mediated isothermal AMPlification (LAMP)	Ref	Ref	Ref	Ref

## Discussion and conclusion

RDTs need quality control especially in low transmission settings because they miss infections at low parasite density (sub-patent) and give false positives up to a month after parasite clearance [18; 7]. This is of importance in a low transmission setting where sub-patent infections make up the majority of the cases (up to 80%). The missed sub-patent infections could sustain malaria transmission and frustrate elimination efforts [8]. Therefore, PCR and LAMP could be used as quality control tools and for active surveillance in geographical hot spots.

RDTs are a good source of DNA for PCR, LAMP and other molecular techniques [25]. This is of importance in low transmission settings where samples for further research on malaria are difficult to obtain. Use of RDTs as a source of DNA could ensure availability of samples for further research in the fight against malaria. The use of RDTs used by Health Workers as sources of DNA will help to advance malaria research in countries going for elimination. The DNA from RDTs could be used for quality control, genotyping and surveillance for malaria resistance markers [26; 9].

RDTs had the lowest sensitivity with a total of 10 positives. This can be explained by the reported number of cases of RDTs failing to detect sub-patent infections [27]. At parasitaemia below 100parasites/μL, RDTs tend to miss infections due to low sensitivity. PCR using pan primers was more sensitive with 6 more positives than RDTs. The higher sensitivity of PCR can be attributed to the amplification of the detection signal, parasite DNA, allowing detection even at low parasitaemia [7]. However, RDTs and PCR showed no significant difference in the detection of malaria infections. A single round of PCR was comparable to RDTs, other studies have shown that nested PCR will detect significantly more malaria infections than RDTs. The use of nested Cytochrome B PCR, real time varATS PCR and high volume PCR have all been reported to have high sensitivity, up to 1parasite/μl which is comparable to LAMP [23]. A single round of PCR was used as it has a shorter turnaround time for results and is cheaper to run than nested PCR. A single round of PCR is a more practical choice for resource settings where malaria is usually endemic, therefore LAMP was used as the golden standard for quality assurance. The ultra-sensitive HRP2-based Malaria Ag P.f test (Alere) is a novel RDT that is supposed to detect malaria up to 4parasites/μl. However, there have been highly varied reports on the performance of the ultra-sensitive HRP2-based Malaria Ag P.f test RDT, therefore it was not used as a quality control for this study [28,29].

The study aimed to determine if a single round of PCR or LAMP could be used for quality control of RDTs in a low transmission setting. LAMP was the most sensitive technique with a total of 22 positives. Although both LAMP and PCR both amplify the detection signal (parasite DNA), LAMP is not affected by most PCR inhibitors (robust) and has higher amplification efficiency. This explains why LAMP was more sensitive than PCR [30]. One round of PCR was run and this explains the difference between PCR and LAMP.

LAMP has been reported to have nested PCR (nPCR) level diagnostic accuracy in a 6^th^ of the turnaround time for results with nPCR [31]. In addition, LAMP is cheaper to set up in terms of machinery and requires minimal training, making it more accessible than nested PCR in lower resource settings [32]. Furthermore, LAMP could have potential uses in identification of malaria hotspots with low density infections that possibly sustain transmission, therefore aiding in efforts to eliminate malaria.

The use of molecular tools as quality control for RDTs in low transmission settings is necessary as the RDTs often miss sub-patent infections which make up the majority of cases in low transmission settings. The malaria cases that are missed by RDTs act as reservoirs that could potentially spread malaria. These asymptomatic cases can also become symptomatic attacks with time. LAMP is better suited for malaria diagnosis with the diagnostic accuracy of nested PCR combined with a cheaper set up, short turnaround time for results and minimal training. RDTs can be used as a DNA source for further research; this makes samples more readily available without the additional cost and effort of collecting Dry Blood Spots (DBS). In order to reach the goal of eliminating malaria, highly sensitive tools need to be employed to make sure that all remaining cases are identified and treated.

## Supporting information

S1 Table PCR and LAMP study data from the study. (XLSX)
